# Contralateral mononostril endoscopic chopsticks technique for an intracavernous epidermoid cyst. Technical case report and systematic review of the literature

**DOI:** 10.1016/j.bas.2026.105980

**Published:** 2026-02-17

**Authors:** Luca Ferlendis, Arianna Fava, Jonathan Chainey, Thibault Passeri, Jerold Justo, Sebastien Froelich

**Affiliations:** Department of Neurosurgery, Lariboisière Hospital, Assistance Publique – Hopitaux de Paris, University of Paris, Paris, France

**Keywords:** Cavernous sinus, Epidermoid cyst, Chopsticks, Endoscopic endonasal, Parasellar, Mononostril

## Abstract

**Introduction:**

Pure intracavernous epidermoid cysts (pIECs) are rare pathologies developing within the cavernous sinus (CS), typically displacing the internal carotid artery medially and cranial nerves laterally. When symptomatic, surgical treatment is indicated, and the approach must be tailored to anatomy.

**Research question:**

Which surgical approach best balances morbidity with the complex anatomy of the CS in pIECs, and can anatomical classification guide surgical planning?

**Material and methods:**

We report the resection of a left pIEC via a contralateral mononostril endoscopic endonasal approach using the Endoscopic Chopsticks Technique (ECsT). A systematic review of the literature was conducted to contextualize the case.

**Results:**

A 23-year-old man with progressive left oculomotor nerve palsy due to a pIEC underwent contralateral mononostril endoscopic surgery using the ECsT. Complete cyst content removal with partial capsule resection was achieved because of adherence to CS neurovascular structures. Sphenoid sinus cranialization and rostral mucosal suture were performed. The postoperative course was uneventful, with recovery of oculomotor deficit and no recurrence at one year. This represents the sixth reported case of a pIEC. The literature review identified 53 cases from 18 articles: transcranial approaches were used in 92.5% of cases, extended endonasal and transorbital approaches in 3.8% each. Partial capsule resection was performed in 58.5% of cases and in 66.6% of pIECs.

**Conclusion:**

Epidermoid cysts of the CS should be treated when symptomatic, with surgical strategy guided by anatomical classification. In selected pIECs, a contralateral mononostril ECsT offers a minimally invasive option that minimizes morbidity while ensuring effective cyst control.

## Highlights

• Pure intracavernous epidermoid cysts represent an extremely rare surgical entity.
• Anatomical classification is key to anticipate nerve displacement and guide the surgical approach.
• Contralateral mononostril ECsT enables target access with minimal endonasal morbidity.
• Total resection is often unsafe; planned subtotal capsule resection may be preferable.

## Abbreviations:

CISS: Constructive Interference in Steady State
CN: cranial nerve
CPA: cerebellopontine angle
CS: cavernous sinus
CSF: cerebrospinal fluid
DWI: diffusion-weighted imaging
ECsT: Endoscopic Chopsticks Technique
EEA: endoscopic endonasal approach
ICA: internal carotid artery
L-ON: left optic nerve
L-VI: left sixth cranial nerve
LWCS: lateral wall of the cavernous sinus
MC: Meckel's cave
MCF: middle cranial fossa
MRI: magnetic resonance imaging
NS: nasal septum
pIEC: pure intracavernous epidermoid cyst
RM: rostral mucosa
seTOA: superior eyelid transorbital approach
SRS: stereotactic radiosurgery
SS: sphenoid sinus
US: ultrasound

## 1 Introduction

Epidermoid cysts are rare, slow-growing lesions that account for 0.2% to 1.8% of all intracranial tumors (Ramesh et al., 2025). Common locations include the cerebellopontine angle (CPA), prepontine cistern, parasellar area, and middle cranial fossa (MCF)(Patibandla et al., 2016; Schiefer and Link, 2008).

Epidermoid cysts originating in the cavernous sinus (CS) are exceedingly rare, and current evidence on their surgical treatment remains limited (Zhou et al., 2018; Gharabaghi et al., 2005; El-et al., 1992; Wang et al., 2013).

These lesions are thought to arise from ectodermal cell rests that persist abnormally after neural tube closure (Netsky, 1988). During embryogenesis, such cells may become sequestered within the meningeal layers surrounding the cranial nerves in the lateral wall of the CS, later giving rise to epidermoid cysts (Wang et al., 2013).

Gharabaghi et al. (2005) classified epidermoid cysts of the CS in 3 different categories: extracavernous, intercavernous and intracavernous. Extracavernous lesions originate outside the CS and secondarily invade or compress it. Interdural cysts arise within the lateral wall of the CS and are located between its two dural layers. Pure intracavernous epidermoid cysts (pIEC) develop inside the CS deforming the normal anatomy and neurovascular relationships often encasing or displacing the internal carotid artery (ICA) medially and pushing the cranial nerves laterally.

Surgical treatment of tumors involving the CS and MCF has significantly evolved during the last decades but remains challenging due to intricate neurovascular anatomy (Jeon et al., 2019; Abiri et al., 2023; Dolenc and Rogers, 2009).

The approach selection should be tailored to each patient to reduce the risk of cranial nerves and vascular injuries while achieving maximal safe resection, with the goal of symptom relief and reduced approach-related morbidity.

In the literature, only a few cases of epidermoid cysts involving the CS have been reported, and even fewer represent pIEC (Gharabaghi et al., 2005; Kline and Galbraith, 1981; Aldea et al., 2022; Wu et al., 2022; Candanedo et al., 2022). Therefore, selecting the optimal approach often remains challenging.

We report our experience in treating a pIEC via a contralateral mononostril approach with the Endoscopic Chopsticks Technique (ECsT)(Labidi et al., 2018; Ferlendis et al., 2025). Closure was performed with hemicranialisation of the sphenoid sinus (SS) and rostral mucosal suture (Di Russo et al., 2021; Alcântara et al., 2025). To our knowledge, this represents the first reported case performed with such a minimally invasive technique.

Additionally, we conducted a systematic review of the literature on surgically treated CS epidermoid cysts, aiming to better standardize their anatomical classification and define the most appropriate surgical strategy for this rare, benign but challenging pathology.

## 2 Material and methods

### 2.1 Technical case report

#### 2.1.1 Clinical presentation and imaging

A 23-year-old man presented to our attention for intermittent diplopia. The initial magnetic resonance imaging (MRI) revealed a left cavernous sinus lesion showing diffusion restriction on diffusion-weighted imaging (DWI) and T1 hypointensity without contrast enhancement, highly suggestive of an epidermoid cyst. Given the surgical risks associated with cavernous sinus pathology, an initial conservative, observational strategy was adopted.

**Fig. 1:**
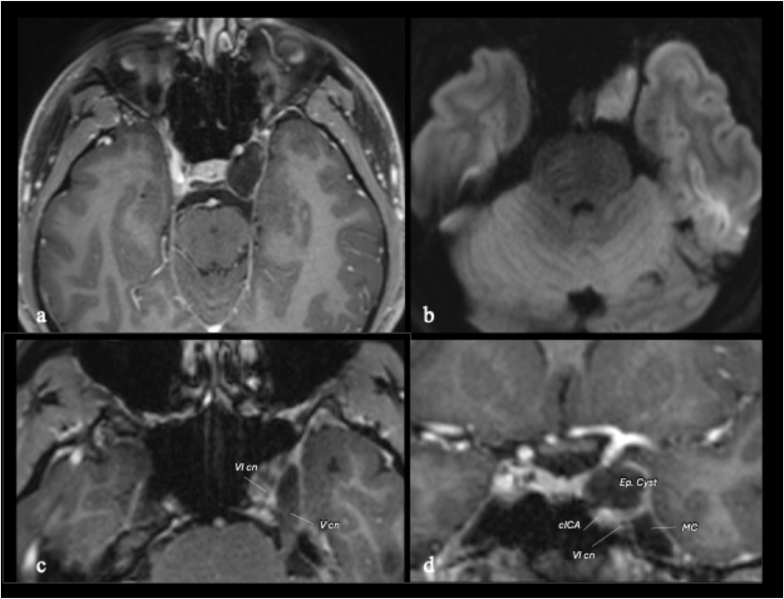
Preoperative axial MRI demonstrating a lesion located in the superolateral compartment of the cavernous sinus, hypointense on T1-weighted images and without contrast enhancement (a), associated with marked diffusion restriction on diffusion-weighted imaging (DWI) (b), findings highly suggestive of an epidermoid cyst. (c, d) Axial and coronal T1-weighted contrast-enhanced images showing inferolateral displacement of the abducens nerve (VI cn) with respect to the cavernous internal carotid artery (cICA). Meckel's cave (MC) is not invaded by the epidermoid cyst.

During follow-up, the patient developed worsening diplopia caused by a left third cranial nerve palsy, with marked limitation of related movements of the left eye and progressive ptosis. Repeat MRI demonstrated progressive enlargement of the lesion (Fig. 1).

**Fig. 2:**
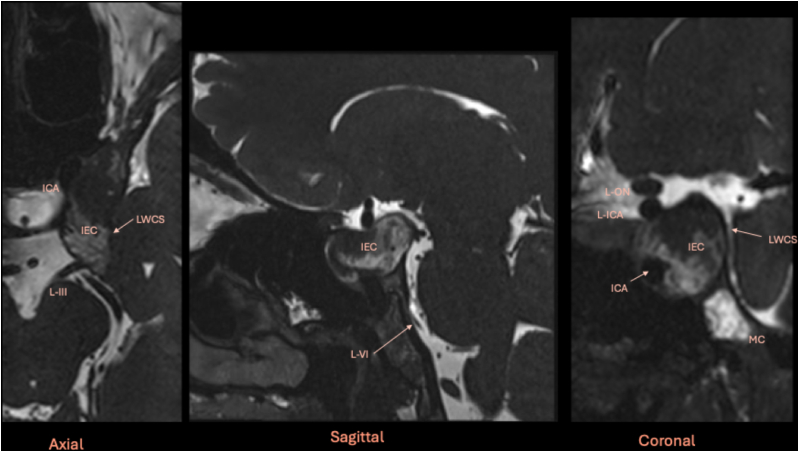
Preoperative multiplanar MRI study with CISS sequences. The pure intracavernous epidermoid cyst (IEC) is clearly located within the cavernous sinus (CS), displacing the internal carotid artery (ICA) anteromedially and laterally bowing the lateral wall of the CS (LWCS), which contains the left third cranial nerve (L-III). *Abbreviations*: ICA: internal carotid artery; MC: Meckel's cave; L-ON: left optic nerve; L-VI: left sixth cranial nerve.

**Fig. 3:**
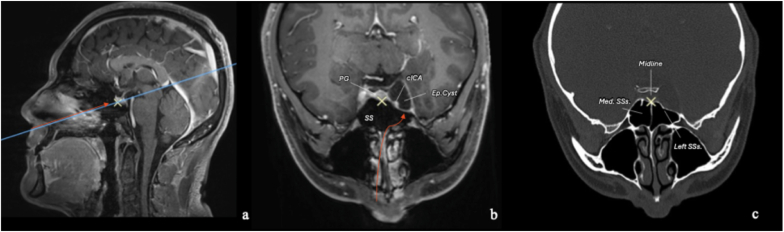
Preoperative radiological planning of the contralateral mononostril endonasal approach. Planning was performed using Carestream Image Suite software, with spatially synchronized images. The blue line in **(a)** indicates the orientation of images **(b)** and **(c)**. The yellow cross is co-registered across all three images and identifies the pituitary gland (PG). The orange arrow in **(a)** and **(b)** indicates the planned surgical trajectory. The cyst creates a natural surgical corridor within the lateral compartment of the cavernous sinus (CS), between the cavernous internal carotid artery (cICA) and the lateral wall of the CS. **(c)** A CT scan in the bone window is essential for understanding sphenoid sinus (SS) anatomy. In this case, the median septation identifies the sella and thus the midline, while a smaller left septation (*better visualized in*Fig. 6) inserts onto the left ICA prominence. Based on this anatomy, a contralateral transrostral approach to access the left CS can be performed without the need for a complete sphenoidotomy, preserving the right sphenoid sinus.

Considering the clinical deterioration, surgical intervention was indicated with the primary objective of third cranial nerve decompression. Based on the location in the upper lateral and superior compartment of the CS (Xu et al., 2024; Fernandez-Miranda et al., 2017), with medial displacement of the ICA and lateral displacement of the cranial nerves, as demonstrated in Fig. 1 and on Constructive Interference in Steady State (CISS) MRI sequences (Fig. 2), a contralateral mononostril endonasal approach was selected (Fig. 3). This technique was chosen to minimize the surgical corridor, avoid brain retraction, and reduce overall operative morbidity, while allowing direct access to the lesion to decompress the oculomotor nerve.

#### 2.1.2 Surgical technique

**Fig. 4:**
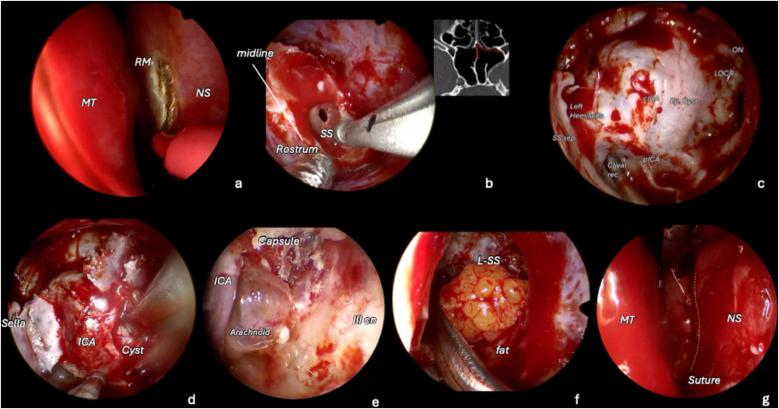
Procedure illustrated through key surgical steps. a) A vertical mucosal incision is made between the posterior nasal septum (NS) and the rostral mucosa (RM). The septum is mobilized, followed by bilateral submucosal dissection. b) The left portion of the rostrum is drilled to access the left sphenoid sinus (SS). c) Visualization of intrasphenoidal landmarks, with the cavernous ICA displaced anteromedially. After performing a parasellar “mini craniotomy,” the dura mater is incised (d), the cyst capsule is opened, and internal decompression is achieved through progressive aspiration while alternating angled endoscopes to optimize visualization and resection. e) Endoscopic exploration of the cavity reveals areas where the capsule is densely adherent to the lateral wall of the cavernous sinus and to the third cranial nerves; therefore, complete capsule removal is not attempted. f, g) Closure is performed by filling and cranializing the left hemi-sphenoid sinus with abdominal fat, followed by suture of the rostral mucosa.

The surgical procedure is illustrated step-by-step in **Supplementary Video 1** and in Fig. 4.

Supplementary data related to this article can be found online at https://doi.org/10.1016/j.bas.2026.105980

The following are the Supplementary data related to this article:

Multimedia component 2 Multimedia component 2

The patient was positioned supine with the head secured in a Mayfield head holder, slightly flexed and rotated toward the surgeon's axis, with mild lateral inclination. To reduce venous congestion, the thorax was elevated. Standard equipment included a neuronavigation system, an endoscopic Doppler ultrasound (US) probe, and neurophysiological cranial nerve (CN) monitoring (CNIII and VI). Angled endoscopes (30°, 45°, 70°) and malleable rotating suctions were used throughout the ECsT.

**Fig. 5:**
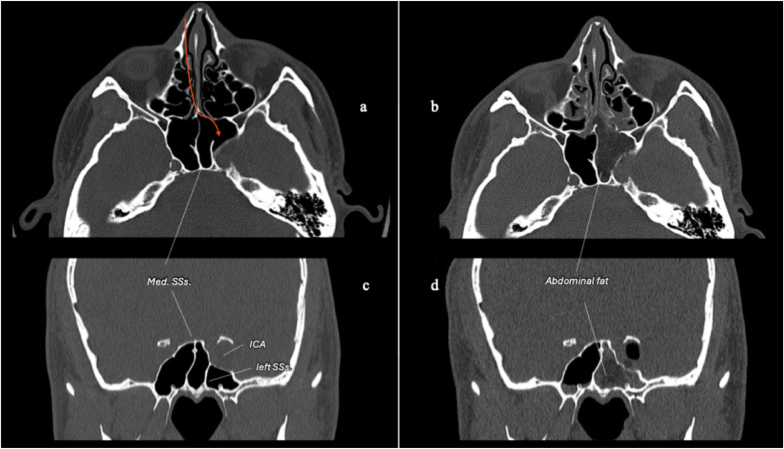
Preoperative **(a, c)** and postoperative **(b, d)** axial and coronal CT scans in the bone window demonstrating the right contralateral mononostril endonasal approach to the left cavernous sinus (CS). **(a, c)** The median sphenoid sinus (SS) septation lies in the midline, while a left SS septation inserts onto the left cavernous internal carotid artery (ICA) prominence. These septations represent important intrasphenoidal landmarks. **(b, d)** Through a left transrostral corridor, only a left hemi-sphenoidotomy is performed. The parasellar mini-craniotomy is reconstructed, the left hemisinus is filled with abdominal fat, and the rostral mucosa is sutured. Nasal anatomy is preserved as much as possible. *Abbreviations:* Med. SSs., median sphenoid sinus septation; SSs., sphenoid sinus septation.

The nasal mucosa was decongested using epinephrine-soaked cottonoids, and the nasal cavity was prepared with a povidone-iodine solution. The abdomen was prepped for fat harvesting. After lateralization of the middle turbinate, a 1,5 cm vertical mucosal incision was made along the posterior septum and rostrum (Fig. 4a). The septum was gently mobilized, followed by bilateral submucosal dissection of the rostrum. The left portion of the rostrum was then drilled to access the left hemi-sphenoid sinus (Fig. 4b). A median sphenoidal septation was present (Fig. 3); therefore, a complete sphenoidotomy was avoided to preserve contralateral nasal drainage and function. A left sphenoidotomy was performed, and a left sphenoid sinus septation inserting onto the left ICA prominence served as an important anatomical landmark (Fernandez-Miranda et al., 2009) (Fig. 5).

The mucosa of the left sphenoid sinus was removed, and the left ICA and tumor bulging into the sinus were identified (Fig. 4c). The parasellar bone was drilled to create a small parasellar “mini craniotomy”, allowing access to the lesion. The ICA was precisely located using the US Doppler probe, and the dura was opened lateral to the artery. Following cranial nerve identification with intraoperative stimulation, the cyst capsule was incised (Fig. 4d), and internal decompression was performed using progressive aspiration while alternating angled endoscopes to optimize visualization and resection.

Endoscopic exploration of the cavity within the superolateral compartment of CS revealed areas of dense capsular adherence to the superior compartment and lateral wall (Fig. 4e). Therefore, complete capsule removal was intentionally avoided to prevent cranial nerve injury. The oculomotor nerve was visualized within the CS superolateral compartment, intact and adequately decompressed. In the event of a minimal cerebrospinal fluid (CSF) leak occurring at the level of the oculomotor porus, abdominal fat was placed at the dural opening. The mini-parasellar bony window was repositioned, and the left sphenoid sinus was cranialized and packed with fat (Fig. 4f). Finally, the rostral mucosa was sutured, reinforced with fibrin glue, and incision was covered with a silastic splint (Fig. 4g). The technique was previously described in other papers from our group (Ferlendis et al., 2025; Di Russo et al., 2021; Alcântara et al., 2025).

### 2.2 Review of the literature

A systematic review was conducted according to PRISMA 2020 guidelines to identify all previously published cases of epidermoid cysts involving the CS. PubMed and Scopus were searched from inception to October 2025 using the terms “epidermoid cyst,” “epidermoid,” “cavernous sinus,” “intracavernous,” “parasellar,” “endoscopic,” and “microsurgical” (e.g., epidermoid cyst AND cavernous sinus).

#### 2.2.1 Eligibility criteria and search strategy

Studies were included if they reported surgically treated epidermoid cysts of the CS, with or without extension into Meckel's cave or the posterior cranial fossa. Eligible papers were case reports, case series, original articles, and reviews focusing on surgical management. Exclusion criteria were lack of surgical treatment; no full English text; unclear or incomplete anatomical localization; missing imaging or surgical data; and isolated Meckel's cave epidermoid cysts. One reviewer (L.F.) screened all records and performed full-text assessment. Surgical videos and imaging were reviewed when available to refine anatomical classification.

**Fig. 6:**
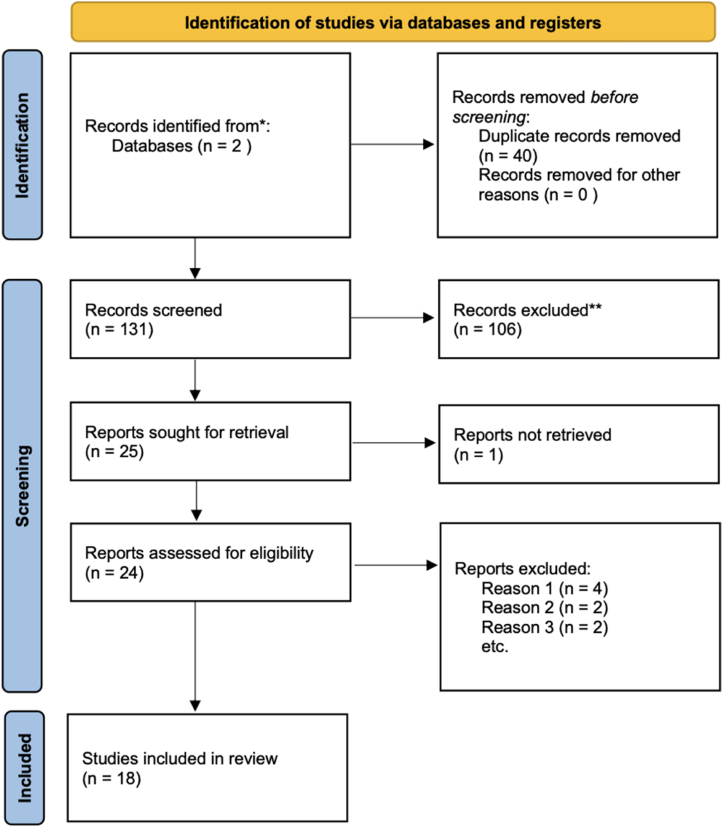
PRISMA 2020 flowchart summarizing the inclusion and exclusion criteria for this systematic review. Reasons for exclusion: Reason 1 = Not in English (n = 4); Reason 2 = Incomplete surgical or imaging data (n = 2); Reason 3 = Unclear anatomical localization (n = 2).

Fig. 6 shows the flowchart according to the PRISMA statement.

#### 2.2.2 Data extraction, risk of bias assessment and classification

Extracted variables included patient demographics, clinical presentation, imaging characteristics, anatomical location, surgical approach, extent of resection, complications, outcomes, and follow-up. Due to the rarity and heterogeneity of cases, results were summarized descriptively. Given the predominance of case reports and small series, no formal risk-of-bias tool was applied; potential bias was addressed through descriptive analysis.

Two authors (L.F., A.F.) independently verified extracted data and classified lesions when possible, according to Gharabaghi et al. (2005) into intracavernous, interdural, or extracavernous types. Discrepancies were resolved by discussion with a third author (T.P.).

## 3 Results

### 3.1 Clinical outcome

The postoperative course was uneventful, and no new neurological deficits occurred. The patient experienced progressive improvement of both diplopia and ptosis. Postoperative management included avoiding the flat position for 10 days, early mobilization, and delayed imaging, performed on postoperative day five to prevent prolonged supine positioning.

**Fig. 7:**
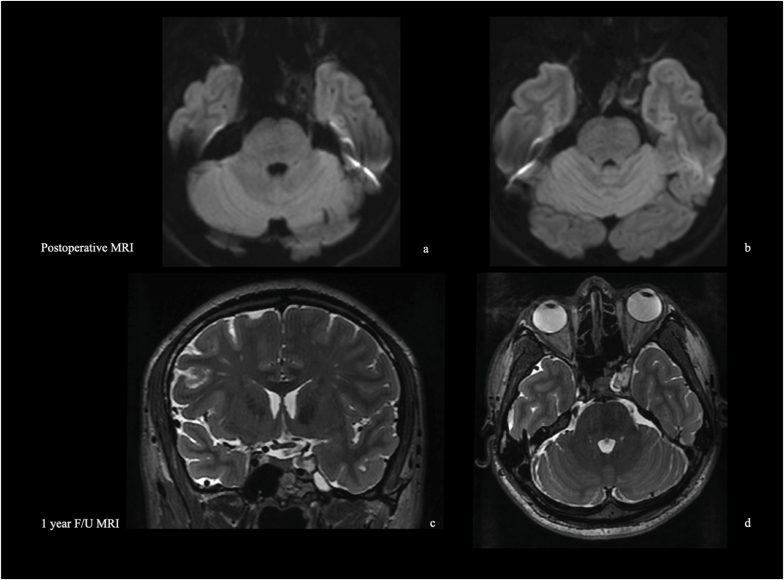
Postoperative MRI demonstrating total cyst removal with subtotal capsule excision in the superoposteromedial portion of the lateral wall of the cavernous sinus (LWCS). The immediate postoperative DWI MRI (a, b) shows no diffusion restriction except for a minimal focal signal corresponding to the capsule remnan**t** (b). At the 1-year follow-up, T2-weighted MRI in coronal and axial planes (c, d) reveals decompression of the cavernous sinus and a signal intensity isointense to CSF, indicating absence of recurrence.

Postoperative MRI confirmed total cyst content removal (Fig. 7). A 48-h antibiotic prophylaxis was administered, and no CSF leak was observed. The patient was discharged on postoperative day five after radiological confirmation. The silastic stent was removed at two weeks, with complete healing of the mucosal suture.

During follow-up, ptosis fully resolved and diplopia continued to improve. The 1-year MRI demonstrated no recurrence and satisfactory decompression of the cavernous sinus (Fig. 7). The signal within the surgical cavity on T2-weighted sequences remained consistent with CSF. Follow-up is ongoing, and stereotactic radiosurgery (SRS) may be considered if recurrence is detected on follow-up MRI.

### 3.2 Literature review

The literature search yielded 171 records (PubMed 68; Scopus 103). After removal of 40 duplicates, 131 unique records were screened, of which 107 were excluded. Twenty-five full-text articles were assessed; one was not retrievable. Of the remaining 24, eight were excluded (four non-English, two with incomplete surgical or imaging data, two with unclear anatomical localization). Ultimately, 18 studies comprising 53 surgically treated patients were included.

#### 3.2.1 Demographic and clinical presentation

The extracted data are summarized in Table 1.

**Table 1:** Systematic review of the literature on surgically treated epidermoid cysts of the cavernous sinus.

Authors (year)	NoC	Sex, age	Symptoms	Side	Location	Classification	Approach	EoR^a^	Outcome	Complications	Long-term outcome	F/U	Recurrence
(Kline and Galbraith, 1981)	1	M, 51	HA, diplopia, III and VI palsy, ptosis, facial hypesthesia	R	CS lateral wall	Extracavernous	Frontotemporal, intradural	Partial capsule resection	HA and hypesthesia improved	Complete R III N palsy	N III palsy recovered, N V1 dysfunction resist	6 m	No
(El-et al., 1992)	2	F, 24	Diplopia, ptosis, IV and III n palsy	R	CS	Interdural	1st op: frontotemporal, intradural; 2nd op: frontotemporal, extradural-intradural	Partial capsule resection	Improved	No	Improved	30 m	No, yes (2 yrs after 1st op); no (after 2nd op)
		M, 53	HA, visual disturbance, diplopia, TN, N IV and III palsy	R	CS	Interdural	Frontotemporal	Partial capsule resection	Improved	No	Improved	/	Yes
(Ikezaki et al., 1992)	1	M, 6	N VI palsy	L	CS	Intracavernous	Frontotemporal (extradural Dolenc anterolateral)	Partial capsule resection	Unchanged	No	Improvement	4 m	No
(Tatagiba and Samii, 2000)	1	M, 68	Right ocular palsy, periorbital pain, pain in the right N V1 branch, hypesthesia of the N V2	R	CS	Interdural	Frontotemporal	Partial capsule resection	Totally recovered	No	Unchanged	24 m	No
(Gharabaghi et al., 2005)	1	F, 27	HA, facial hypesthesia	R	CS, MC	Intracavernous	Frontotemporal, intradural	Total	Unchanged	No	Improvement	12 m	No
(Bonde and Goel, 2008)	1	F, 45	TN, facial hypesthesia, TM atrophy, absent corneal reflex	L	CS, MC	Interdural	Subtemporal extradural	Total	Pain resolved	No	Unchanged, still hypoesthesia	36 m	No
(Ghaemi et al., 2010)	1	M, 71	Diplopia, III n palsy	R	CS	Interdural	Frontotemporal	Partial capsule resection	Aggravation N III palsy	Yes transient	Recovered	18 m	Yes
(Chung et al., 2012)	1	M, 21	HA, diplopia, visual disturbance, ptosis III palsy	R	CS	Intracavernous	Frontotemporal, extradural, EndoscopicA	Total	Pain resolved, unchanged palsy	No	Unchanged palsy	24 m	No
Ren et al., 2012 (Ren et al., 2012)	1	M, 30	Dizziness, right eye discomfort, right face	R	CS	Interdural	Frontotemporal	Partial capsule resection	Resolved	No	/	/	
(Jhawar et al., 2013)	1	F, 40	Facial hypesthesia, absent corneal reflex, spastic hemiparesis	L	CS, MC	Interdural	Subtemporal extradural	Partial capsule resection	Improved	No	Unchanged	12 m	No
(Wang et al., 2013)	4	F, 32	Visual disturbance, facial hypesthesia	L	CS	Interdural	Frontotemporal, intradural	Partial capsule resection	Improved	No	Numbness	84 m	No
		F, 29	HA, facial hypesthesia, N V palsy	R	CS	Interdural	Frontotemporal, intradural	Total	Improved	No	Hemifacial numbness	48 m	No
		M, 51	HA, ptosis, facial numbness, V and N III palsy	L	CS	Interdural	Frontotemporal, intradural	Partial capsule resection	Improved	No	Unchanged	48 m	No
		F, 50	HA, visual disturbance, IV and III palsy	L	CS	Interdural	Frontotemporal, intradural	Total	Improved	No	Unchanged	60 m	No
Zhou et al., 2017 (Zhou et al., 2018)	31	Age 46.2 ± 14.8; 16M; 15 F	Facial numbness (16), diplopia (11), temporal muscle atrophy (10), VI palsy (9), III palsy (5), VII palsy (2)	/	22 MCF, 9 PCFe	/ 2 interdural 1 Multicompartmental (extracavernous)	Frontotemporal, FTOZ, subtemporal Extradural (18), intradural (10), combined (intradural e retrosygmoid) 2; EndoscopicA (3)	Total: 16, Partial capsule resection 13, “Subtotal” (contents + capsule): 2	Transient oculomotor (3); Unchanged (23); Improved (5)	5 aseptic m, 2 septic meningitis	Recovery oculomotor deficit (2) Lost on F/U (1)	4.6 +-3 y	No
(Sun et al., 2018)	2	M, 61	N III, IV, VI palsy	/	CS, CPA, suprasellar	Multicompartmental (extracavernous)	Frontotemporal extended	“Near Total” (Capsule N/S)	Unchanged	No	/	13 m	No
		F, 18	HA, syncope	/	MC, CS, ITF, orbit	Multicompartmental (extracavernous)	Frontotemporal extended + EEEA (2nd) + Frontotemporal (3rd)	Total after 3rd surgery (Capsule N/S)		Seizure, CSF leak, mild cognitive difficulties, nonorganic hearing loss	/	106 m	Yes after 2nd surgery, no after the 3rd
Aldea et al., 2021 (Aldea et al., 2022)	1	F, 34 yo	Partial N VI palsy, trigeminal dysesthesia	R	CS, MCF, PCFe	Intracavernous	EEEA (Transpterygoid) + NSF	Partial capsule resection	Transient increases of dysesthesia	No	Recovery at 3 m	3 m	No
(Wu et al., 2022)	1	F, 54 yo	Intermittent temporal pain	L	CS Interdural	Intracavernous	eEEA	Partial capsule resection	Improved	No	Unchanged	12 m	No
(Candanedo et al., 2022)	1	F, 22 yo	Partial N III palsy	R	CS Interdural	Interdural	Pterional	Partial capsule resection	Transient III worsening	Yes transient	Recovery at 3 m	No residual	No
(Han et al., 2023)	1	F, 37 yo	HA, hemifacial numbness	R	CS, MC	Interdural	seETOA	Total (Capsule N/S)	Unchanged, chemosis 1 week	No			
(Manogaran et al., 2025)	1	F, 33	HA, hemifacial numbness	R	CS, PCFe	Interdural	seTOA (+OR)	Partial capsule resection	Unchanged	No	Unchanged	3 months	No

Our Case (2025)	1	M, 23	N III partial palsy aggravation	L	CS	Intracavernous	Contralateral ECsT	Partial capsule resection	Improved	No	Improved	1 year	No

Abbreviations: CPA: cerebellopontine angle; CS: cavernous sinus; EEEA: extended endoscopic endonasal approach; seTOA: endoscopic superior eyelid transorbital approach; F: female; HA: headache; L: left; M: male; m: months; MC: Meckel's cave; MCF: middle cranial fossa; N: cranial nerve; NSF: nasoseptal flap; OR: orbital rim osteotomy; PCFe: posterior cranial fossa extension; R: right; TM: temporalis muscle; TN: trigeminal neuralgia.

a Extent of Resection (EoR): *Total* indicates complete removal of cyst contents and capsule; *partial capsule resection* indicates complete evacuation of cyst contents with intentional capsule remnants left in situ. The terms “near-total” and “subtotal,” as used by the original authors, could not be reliably reclassified according to these criteria. *N/S*: not specified.

Fifty-three cases of cavernous sinus epidermoid cysts were identified. The mean age was 43.2 years (range 6–71), with 25 males and 28 females.

The most common symptoms were hemifacial numbness/dysesthesia (25 patients, 47.1%) and diplopia (22 patients, 41.5%). Headache occurred in ten cases (18.8%).

Cranial nerve involvement included: CN III palsy in 14 cases (6 complete, 8 partial), CN IV palsy in 4 (7.5%), CN VI palsy in 13 (24.5%), and temporalis muscle atrophy in 2 patients.

#### 3.2.2 Anatomical distribution and classification

All lesions originated in the CS; four extended into Meckel's cave. The series by Zhou et al. (2018) accounted for 31 cases, with nine extending into the posterior fossa and 22 into the MCF. Two additional cases (Alcântara et al., 2025; Xu et al., 2024) showed posterior fossa extension, and one demonstrated multicompartmental spread including the infratemporal fossa.

Classification according to Gharabaghi was feasible in only 25 of the 53 reported cases: five intracavernous, sixteen interdural, and four extracavernous, three of them with multicompartmental involvement. The remaining 28 lesions could not be classified because of insufficient anatomical details. Although Gharabaghi et al. originally described six intracavernous epidermoid cysts, four cases reported in that series could not be included in the present systematic review because they did not meet the predefined inclusion criteria.

Tumor dimensions were inconsistently reported and are therefore not included in the summary table.

#### 3.2.3 Surgical approaches and extent of resection (EoR)

Most cases were treated via transcranial microsurgical approaches (49 cases, 92.5%).

Endoscopic techniques included extended EEA (2 cases, 3.8%) and superior eyelid transorbital approach (seTOA) (2 cases, 3.8%); four transcranial procedure used endoscopic assistance. Notably, extended EEA was used exclusively for two pIEC, whereas seTOA was applied in two interdural CS epidermoid cysts.

EoR was defined as follows: *total* resection indicated complete removal of both cyst contents and capsule, whereas *partial capsule resection* referred to complete evacuation of cyst contents with intentional capsule remnants left in situ based on intraoperative judgment. Capsule status was not specified in three cases (N/S).

According to these criteria, partial capsule resection was reported in 27 cases. In three additional cases, the EoR could not be reliably reclassified due to insufficient detail in the original reports; these were described by the authors as “near-total” resection (one case, capsule status N/S) or “subtotal” resection involving both cyst contents and capsule (two cases).

Total resection was reported in 23 cases: 20 after the initial surgery, one following a third surgical procedure for recurrence, and two cases in which total resection was stated but capsule status was N/S. Overall, complete capsule removal was not achieved in 31 of 53 cases (58.5%).

Correlation between EoR and anatomical classification was possible only in a subset of cases.

In pIECs total resection was accomplished in 2 of 5 cases (40%), whereas partial capsule resection was performed in the remaining three (60%). Among interdural cases (16), partial capsule resection was performed in 10 cases and total resection in 6. Extradural lesions (4) were difficult to classify according to EoR and were more frequently associated with non-total resection.

Recurrence requiring reoperation was reported in one case at 24 months, while another patient underwent sequential endonasal and transcranial procedures for late recurrence.

#### 3.2.4 Postoperative outcomes and complications

Reported complications in the 53 patients included: complete persistent CN III palsy (1), transient oculomotor deficits with recovery during follow-up (5), transient dysesthesia (1), aseptic meningitis (5), septic meningitis (2), seizure (1), CSF leak (1), mild cognitive disturbance (1), and nonorganic hearing loss (1), chemosis in seTOA (1).

Overall, fourteen patients showed improvement or resolution of preoperative symptoms. Neurological improvement was more frequently observed in interdural lesions. Among patients treated with total resection, no postoperative neurological deterioration was reported; three patients improved, one remained clinically stable, and postoperative outcome was not specified in one case. In interdural lesions treated with partial capsule resection, no permanent neurological worsening occurred: eight patients improved or remained stable, while two experienced transient third cranial nerve palsy with complete recovery at three months.

In pIECs, among the two cases treated with total resection, hemifacial pain resolved in one patient, while no improvement or worsening of preoperative oculomotor nerve deficits was observed. In cases managed with partial capsule resection, the clinical outcome remained unchanged, with one patient experiencing a transient increase in facial dysesthesia; oculomotor deficits remained unchanged in the remaining cases.

Extradural epidermoid cysts were more frequently associated with postoperative surgical or medical complications.

#### 3.2.5 Follow up and recurrence

The mean follow-up duration was 35.8 months (range, 3–106 months). Recurrence was reported in four cases (7.5%). In two patients, recurrence occurred at 18 and 24 months, respectively. In the remaining two cases, reoperation was performed, with no further recurrence observed during subsequent follow-up. All other cases demonstrated stability or absence of residual lesion at last follow-up.

## 4 Discussion

Epidermoid cysts of the CS represent a rare subset of intracranial epidermoid (Ramesh et al., 2025). Their deep position and close relationship with the ICA and cranial nerves make their surgical treatment challenging. In our systematic review, only 53 surgically treated cases were identified over more than four decades, underscoring the rarity of these lesions and the lack of large clinical series.

Clinical presentation reflects the complex neuroanatomy of the CS, and progression of cranial nerve dysfunction often determines the timing of surgery, as in our patient. MRI remains essential for diagnosis, with diffusion restriction on DWI being highly suggestive of epidermoid cyst (Zhou et al., 2018; Ren et al., 2012). However, heterogeneous imaging quality and reporting frequently limit precise anatomical classification. Our case represents only the sixth clearly documented pIEC in the literature.

pIECs differ fundamentally from interdural or extracavernous lesions, as they develop entirely within the CS and are closely related to cranial nerves, venous structures, and the cavernous segment of the ICA. This distinction is critical, since anatomical classification directly influences surgical strategy and the EoR.

Because these lesions are benign yet situated in a critical location, surgery is generally reserved for symptomatic progressive cases. Although no universally accepted classification system exists, reproducible anatomical classifications remain essential for surgical planning. The systems proposed by Gharabaghi and El Kalliny (El-et al., 1992) are particularly useful, as they predict cranial nerve displacement patterns and the relationship between the cyst and the parasellar ICA, two key factors influencing surgical corridor selection. High-resolution MRI sequences such as CISS, and 3D T1-weighted imaging further refine preoperative assessment. In our case, analysis of nerve entry angles into the CS allowed accurate identification of a true intracavernous lesion and guided selection of the surgical approach. Notably, such advanced imaging was inconsistently reported in previous series.

Moreover, CS epidermoid cysts arise from ectodermal remnants within the meningeal layers of the CS (Wang et al., 2013; Tatagiba and Samii, 2000). For this reason, a pathology-oriented classification based on CS walls may be more predictive than proposed subcompartmental anatomy of the CS (Xu et al., 2024; Fernandez-Miranda et al., 2017) in relation to the ICA when planning surgical strategy in these lesions. However, detailed preoperative neuroimaging analysis and localization of the pIEC in relation to the ICA using subcompartmental anatomy may still clarify the regional neurovascular relationships.

In contrast, the classification proposed by Zhou (Zhou et al., 2018), based mainly on posterior fossa extension, appears less predictive of surgical strategy. The large series by Zhou et al. mainly included large, lateral, and often multicompartmental lesions, for which a precise distinction between pure intracavernous, interdural, and extracavernous origin was not consistently possible. Accordingly, these cases were not considered true pIECs according to strict Gharabaghi criteria.

From a surgical perspective, pure intracavernous lesions, characterized by anteromedial displacement of the ICA and lateral displacement of cranial nerves, may be most safely approached through an anterior endonasal route. Conversely, interdural or extracavernous lesions that displace cranial nerves medially appear better suited for anterolateral or lateral transcranial approaches, or for selected transorbital routes (El-et al., 1992; Jeon et al., 2019; Han et al., 2023). Similar conclusions have been reported in a recent systematic review of dermoid cysts (Ros et al., 2026), which, although histologically distinct, share a comparable embryological origin and growth behavior.

The main surgical limitation in pIECs remains the dense adherence of the capsule to the contents of the CS, including cranial nerves, venous structures, and the intracavernous ICA with its branches. This anatomical constraint often precludes complete capsule removal without incurring significant neurological morbidity. Accordingly, in our review, complete capsule removal was not achieved in 58.5% of cases, reflecting a strategy of maximal safe resection rather than aggressive capsule dissection.

When focusing specifically on pIECs, this tendency was even more pronounced. Total resection was feasible in only a minority of cases (two of six, included our case), whereas partial capsule resection was required in most patients (four of six cases). Importantly, complete capsule removal in pIECs did not consistently translate into superior neurological outcomes, particularly when oculomotor deficits were already present preoperatively.

In this context, the commonly used term “subtotal resection” may be misleading, as it implies a quantitative assessment that is not applicable to capsule removal in epidermoid cyst surgery. We therefore adopted the term *partial capsule resection* to describe intentional capsule remnants left in situ. Our findings further support the role of anatomical classification in predicting both EoR and neurological outcome: interdural lesions more often allow safer capsule dissection and show more favorable postoperative recovery, whereas pIECs are frequently associated with stable or limited neurological improvement due to intrinsic anatomical constraints.

In our case, the growth pattern within the superolateral compartment of the CS created a tumor-induced surgical corridor between the ICA and the lateral wall, allowing safe direct access without carotid mobilization. The contralateral mononostril EEA with the ECsT enabled effective maneuverability within a narrow corridor and adequate visualization of the operative field. Avoidance of an extended endonasal approach and nasoseptal flap harvesting contributed to preservation of nasal function and further reduction of approach-related nasal morbidity (Ferlendis et al., 2025; Gui and Tham, 2022).

Lesion size also appears to be a relevant factor. Small pIECs, such as the present case, are rare and particularly challenging due to the limited working space and the high risk associated with capsule manipulation. In larger intracavernous lesions, microsurgical approaches may offer superior bimanual control and a wider operative corridor, potentially facilitating safer capsule dissection. EEA may therefore be most appropriate for small, well-selected pIECs in which the primary goal is neural decompression rather than complete capsule removal. In this setting, extensive endoscopic lateral transcavernous exposure is not always mandatory (Xu et al., 2024), and a tailored minimally invasive approach may be sufficient when anatomical conditions are favorable. Nevertheless, approach selection must remain individualized, considering lesion size, extension, and surgeon experience.

Despite the high rate of subtotal capsule resection, recurrence was uncommon (7.5%), a finding that is likely influenced by the relatively short mean follow-up duration of 35.8 months. Given the very slow-growing nature of epidermoid cysts, long-term follow-up remains essential. In selected cases, radiotherapy or SRS may represent a useful adjuvant option in the event of recurrence (Morshed et al., 2019; Verma et al., 2025).

Given the benign natural history of residual epidermoid tissue and the availability of radiosurgical options, a planned partial capsule removal may be an appropriate and safe strategy for pIECs in which the resection of the capsule carries a high risk of cranial nerve injury.

## 5 Limitations

This work comes with several limitations. First, it is based on a single clinical case, and the anatomy of our patient inevitably limits how broadly the observations can be applied. The systematic review we conducted also reflects the rarity of these lesions: most published reports are isolated cases, often heterogeneous in quality, which introduces an unavoidable degree of selection and publication bias. In many articles, imaging or anatomical descriptions were incomplete, making it difficult to apply existing classification systems in a consistent way. Follow up was uneven across studies, which may lead to an underestimation of recurrence. Finally, the feasibility of the endoscopic approach we used depends not only on the lesion's configuration but also on the surgeon's experience and on individual anatomical factors, which can vary considerably from case to case.

In conclusion, meticulous preoperative neuroradiological analysis is essential to accurately predict the surgical corridor and cranial nerve displacement patterns. A high level of expertise in endoscopic skull base anatomy, together with the appropriate use of surgical instrumentation, is fundamental for the safe practice of minimally invasive endonasal surgery and the Chopsticks technique (Ferlendis et al., 2025).

## 6 Conclusions

Epidermoid cysts are rare lesions and, when involving the CS, represent a significant neurosurgical challenge. Surgical treatment should be considered in symptomatic patients with cranial nerve deficits due to compression within the CS. In pIECs, complete capsule removal is frequently limited, making maximal safe resection an appropriate strategy. Selection of the safest surgical approach should be guided by anatomical classification and supported by high-resolution imaging. Intracavernous cysts are better suited to anterior endonasal approaches, whereas interdural or multicompartmental lesions are generally better managed through anterolateral or lateral transcranial routes. In selected cases of pIEC, a contralateral mononostril endonasal approach using the Chopsticks technique may effectively reduce nasal morbidity. Further accumulation of well-documented cases will be necessary to refine the optimal surgical management of these rare lesions.

## Informed consent

Written informed consent for publication of clinical data and images was obtained from the patient.

## Author's contributions

**Luca Ferlendis:** Writing – Original draft, Writing - Reviewing and Editing, Methodology, Video Editing **Arianna Fava**: Conceptualization, Reviewing, Writing - Reviewing and Editing**,** Video Editing**; Jerold Justo**: Visualization; **Jonathan Chainey:** investigation **Thibault Passeri**: Supervision, Visualization; **Sebastien Froelich**: Supervision, Surgeon, Reviewing and Editing.

## Declaration of competing interest

The authors declare that they have no known competing financial interests or personal relationships that could have appeared to influence the work reported in this paper.
