# Supplementary material for: Contralateral mononostril endoscopic chopsticks technique for an intracavernous epidermoid cyst. Technical case report and systematic review of the literature
Source: Brain Spine. 2026 Feb 17;6:105980. doi: 10.1016/j.bas.2026.105980 (PMC12927310; doi:10.1016/j.bas.2026.105980)
Supplement: Multimedia component 1 [file mmc1.docx]

**Structured Review – Methods**

A systematic review was conducted according to PRISMA 2020 guidelines to identify all previously published cases of epidermoid cysts involving the cavernous sinus (CS). PubMed and Scopus were searched from inception to October 2025 using the terms “epidermoid cyst,” “epidermoid,” “cavernous sinus,” “intracavernous,” “parasellar,” “endoscopic,” and “microsurgical” (e.g., epidermoid cyst AND cavernous sinus).

## Eligibility criteria and Search Strategy

Studies were included if they reported surgically treated epidermoid cysts of the CS, with or without extension into Meckel’s cave or the posterior cranial fossa. Eligible papers were case reports, case series, original articles, and reviews focusing on surgical management. Exclusion criteria were lack of surgical treatment; no full English text; unclear or incomplete anatomical localization; missing imaging or surgical data; and isolated Meckel’s cave epidermoid cysts. One reviewer (L.F.) screened all records and performed full-text assessment. Surgical videos and imaging were reviewed when available to refine anatomical classification.

## Data extraction, Risk of Bias assessment and Classification

Extracted variables included patient demographics, clinical presentation, imaging characteristics, anatomical location, surgical approach, extent of resection (EoR), complications, outcomes, and follow-up. Due to the rarity and heterogeneity of cases, results were summarized descriptively. Given the predominance of case reports and small series, no formal risk-of-bias tool was applied; potential bias was addressed through descriptive analysis.

Two authors (L.F., A.F.) independently verified extracted data and classified lesions when possible, according to Gharabaghi et al.^5^ into intracavernous, interdural, or extracavernous types. Discrepancies were resolved by discussion with a third author (T.P.).

The study selection process is summarized in the PRISMA 2020 flow diagram (**Figure X**).

**Figure X.** PRISMA 2020 flowchart summarizing the inclusion and exclusion criteria for this systematic review. Reasons for exclusion: Reason 1 = Not in English (n = 4); Reason 2 = Incomplete surgical or imaging data (n = 2); Reason 3 = Unclear anatomical localization (n = 2).

**
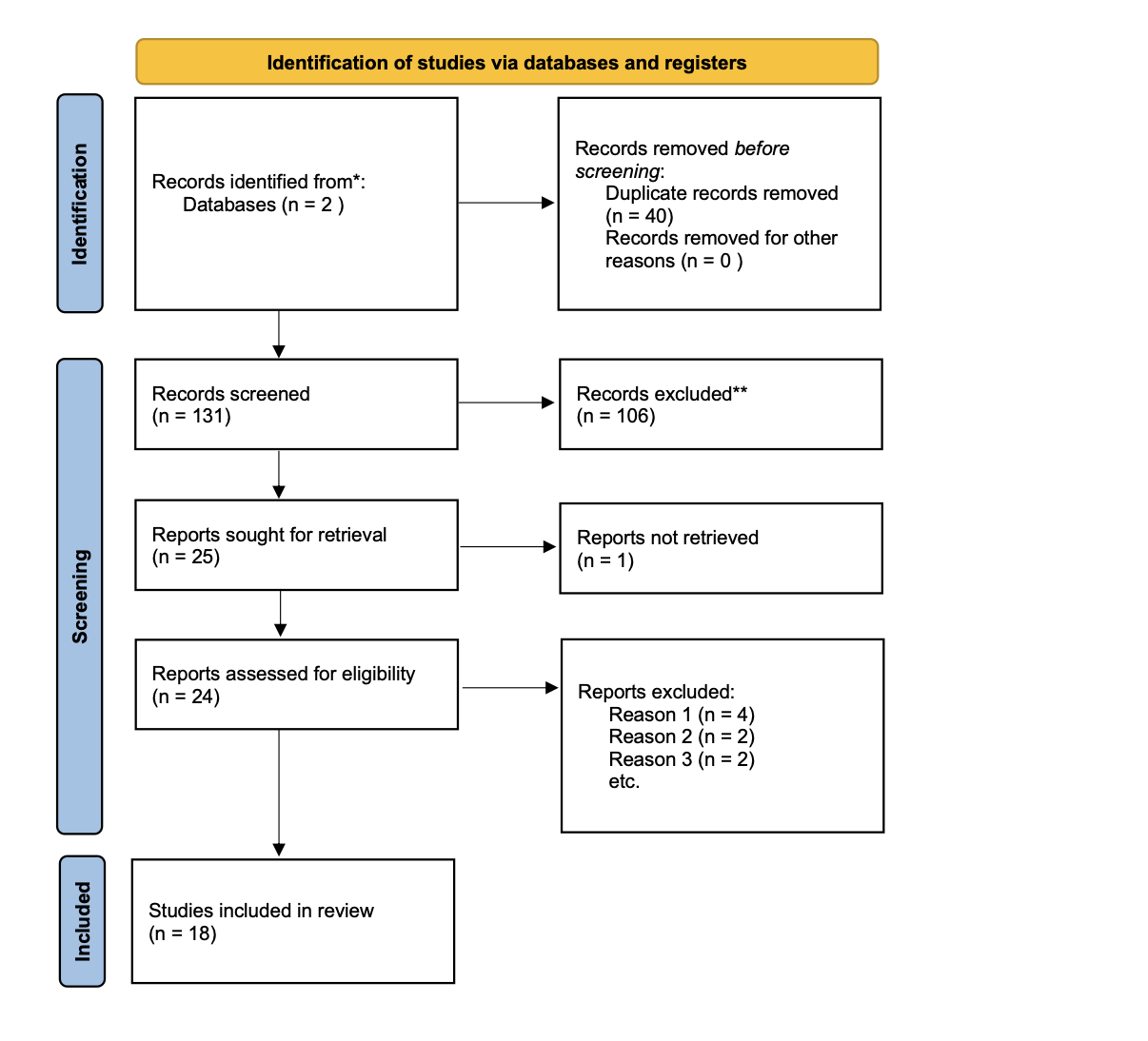
**
